# Supplementary material for: Novel Taxol-Derivative, STO-1, Induces Selective Anti-Tumor Immunity and Sustained Remission of Glioblastoma Without Triggering Autoimmune Reactions
Source: Cells. 2025 Oct 30;14(21):1703. doi: 10.3390/cells14211703 (PMC12607822; doi:10.3390/cells14211703)
Supplement: Supplementary file 1 [file cells-14-01703-s001.zip › cells-3909002-supplementary.pdf]

Supplementary Data

STO-1 Crosses the Blood-Brain Barrier (BBB)

Following tail-vein infusion of STO-1 in liposomes (see Methods) [31], brains from mice were harvested after specific time intervals, homogenized in 70% aqueous acetonitrile (ACN), and the extracts analyzed by high-performance liquid chromatography (HPLC) calibrated against a standard STO-1 curve. Within 15 minutes of infusion, STO-1 was detected in the brain at approximately 400 nM, and was metabolized over time (**Fig. S1**).

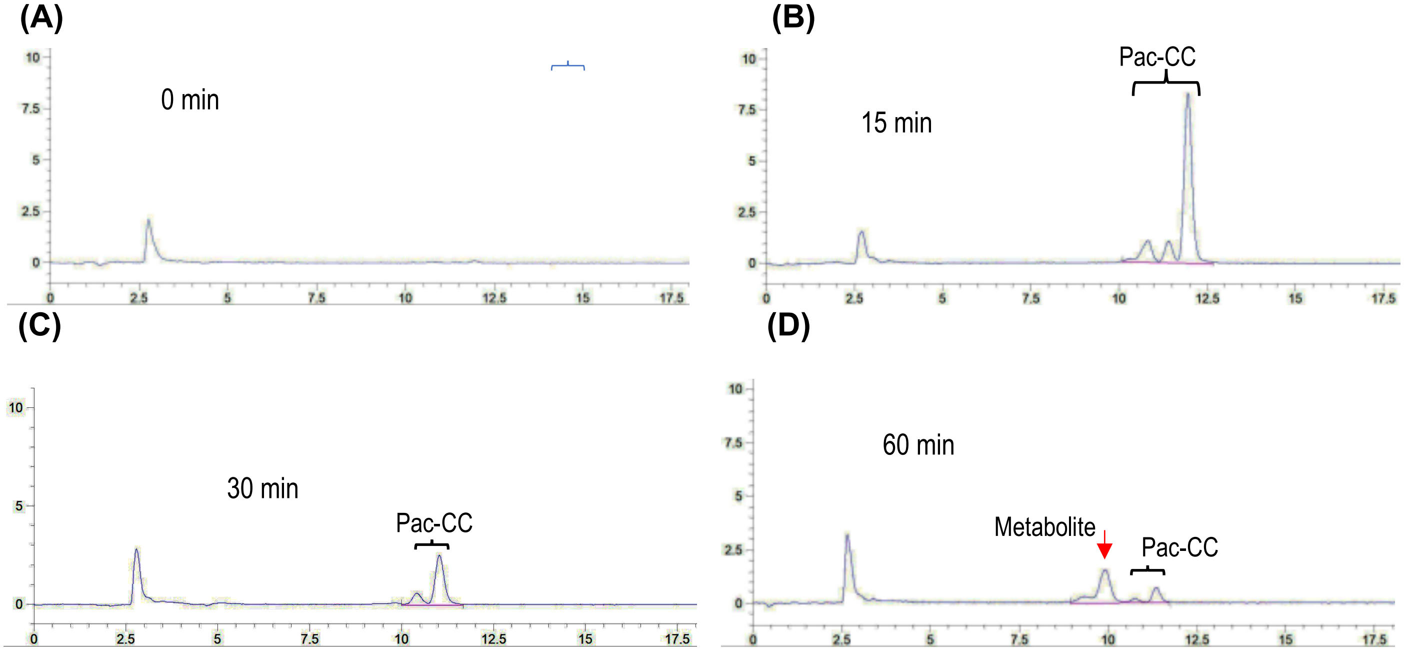

Fig. S1. Following tail-vein injection of liposomal STO-1, it is detected in brain extracts within 15'. *STO-1 is metabolized in 60'* ***(A-D)****. Brain volume = 0.5 ml. Mean STO-1 conc. at 15' = 0.40 μM (0.53 μg/ml).*

STO-1 and Pac Have Similar Potency in Eliminating Mouse Glioblastoma GL261 Cells In Vitro

We evaluated the efficacy of STO-1 in eliminating GL261 cells through IC50 analysis. STO-1 exhibited an IC50 of 20.51 nM, which was not statistically different ($p = 0.42$) from that for paclitaxel alone (46.82 nM) (**Fig. S2**).

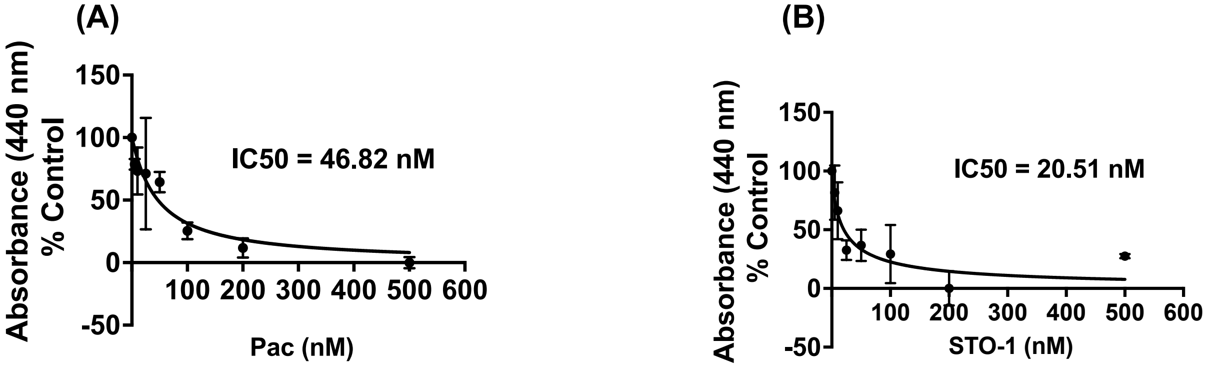

Fig. S2. Both STO-1 and Pac eliminate GL261 cells in vitro. ***(A)*** *Paclitaxel (Pac)*, ***(B)*** *STO-1.*

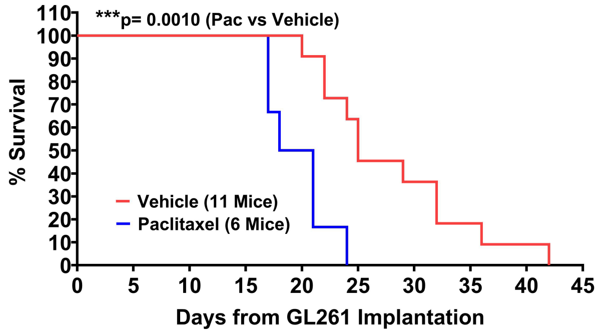

Fig. S3. Long-term Pac treatment does not extend survival of mice from GBM. *Following implantation of 2 x 10^4 GL261 cells on day 1, liposomal Pac or Vehicle treatment was conducted as described in Fig. 9 legend.*
